# Retinal AD Biomarkers in Cognitively Unimpaired Older Adults

**DOI:** 10.1002/alz.089275

**Published:** 2025-01-09

**Authors:** Ashley N. Price, Peter J. Snyder, Jennifer Strenger, Louisa I. Thompson, Megan Stradtman, Stuart Sinoff, Jessica Alber

**Affiliations:** ^1^ University of Rhode Island, Kingston, RI USA; ^2^ Butler Hospital Memory and Aging Program, Providence, RI USA; ^3^ Alpert Medical School of Brown University, Providence, RI USA; ^4^ BayCare Health, Clearwater, FL USA

## Abstract

**Background:**

The retina is a novel biomarker target for examining AD pathology, specifically beta‐amyloid (Aβ). Though relationship between retinal Aβ and standard preclinical AD biomarkers (cerebral Aβ PET) remains understudied, retinal inclusion bodies (IB, strongly hypothesized to contain fibrillar Aβ), predict cerebral Aβ load in the anterior cingulate cortex (ACC). This study explored the value of retinal IB as a preclinical AD biomarker, comparing number and surface area (SA) of IB with cerebral Aβ PET.

**Method:**

36 older adults aged 55‐80 were identified as cognitively unimpaired based on Clinical Dementia Rating Scale (CDR) of 0 and a score ≥24 on the Montreal Cognitive Assessment (MoCA). PET status (20 Aβ+, 16 Aβ‐) was available through prior studies. Retinal IB data was captured on Heidelberg SPECTRALIS using blue light fundus autofluorescence. Number and surface area of IB were computed manually by two qualified raters. Inter‐rater reliability was measured with Cohen’s Kappa. Binomial logistic regression and ROC assessed the accuracy, sensitivity, and specificity of retinal IB in predicting Aβ PET status (positive vs. negative).

**Result:**

Inter‐rater reliability was high (Cohens kappa =0.9). Mann Whitney U Tests showed no significant group differences in the number (U=156.5, p=.906) and SA of IB (U=156.5, p=.962) between the Aβ+ and Aβ‐ PET groups. Binomial logistic regression including predictors (APOE status, age, MoCA score, and SA of IB), showed that SA of IB accurately predicted PET status 61.1% of the time, (sensitivity 75%, specificity 43.8%, AUC =.408). The analysis was repeated with the number of IB, showing that PET status was correctly predicted 63.9% of the time (sensitivity 85%, specificity 37.5%, AUC=.448).

**Conclusion:**

Retinal IB, strongly hypothesized to contain fibrillar Aβ, were not strongly predictive of Aβ PET status in this sample. Given the sample characteristics, the results align with what we hypothesized. Further longitudinal analysis with larger sample sizes and PET centiloid readings in regions of early cerebral Aβ accumulation could help determine the value of retinal imaging as a risk biomarker in preclinical AD.

